# Exclusion of NFAT5 from Mitotic Chromatin Resets Its Nucleo-Cytoplasmic Distribution in Interphase

**DOI:** 10.1371/journal.pone.0007036

**Published:** 2009-09-14

**Authors:** Anaïs Estrada-Gelonch, Jose Aramburu, Cristina López-Rodríguez

**Affiliations:** Immunology Unit, Department of Experimental and Health Sciences (DCEXS), Universitat Pompeu Fabra (UPF), Parc de Recerca Biomèdica de Barcelona (PRBB), Barcelona, Spain; Roswell Park Cancer Institute, United States of America

## Abstract

**Background:**

The transcription factor NFAT5 is a major inducer of osmoprotective genes and is required to maintain the proliferative capacity of cells exposed to hypertonic stress. In response to hypertonicity, NFAT5 translocates to the nucleus, binds to regulatory regions of osmoprotective genes and activates their transcription. Besides stimulus-specific regulatory mechanisms, the activity of transcription factors in cycling cells is also regulated by the passage through mitosis, when most transcriptional processes are downregulated. It was not known whether mitosis could be a point of control for NFAT5.

**Methodology/Principal Findings:**

Using confocal microscopy we observed that NFAT5 was excluded from chromatin during mitosis in both isotonic and hypertonic conditions. Analysis of NFAT5 deletions showed that exclusion was mediated by the carboxy-terminal domain (CTD). NFAT5 mutants lacking this domain showed constitutive binding to mitotic chromatin independent of tonicity, which caused them to localize in the nucleus and remain bound to chromatin in the subsequent interphase without hypertonic stimulation. We analyzed the contribution of the CTD, DNA binding, and nuclear import and export signals to the subcellular localization of this factor. Our results indicated that cytoplasmic localization of NFAT5 in isotonic conditions required both the exclusion from mitotic DNA and active nuclear export in interphase. Finally, we identified several regions within the CTD of NFAT5, some of them overlapping with transactivation domains, which were separately capable of causing its exclusion from mitotic chromatin.

**Conclusions/Significance:**

Our results reveal a multipart mechanism regulating the subcellular localization of NFAT5. The transactivating module of NFAT5 switches its function from an stimulus-specific activator of transcription in interphase to an stimulus-independent repressor of binding to DNA in mitosis. This mechanism, together with export signals acting in interphase, resets the cytoplasmic localization of NFAT5 and prevents its nuclear accumulation and association with DNA in the absence of hypertonic stress.

## Introduction

NFAT5 is a transcription factor that belongs to the Rel family (NFAT and NF-κB) [1], [2]. Its DNA-binding domain is considered a hybrid between that of NFAT and NF-κB proteins, since it binds NFAT-like DNA elements [3] but also conserves the molecular mechanism that mediates dimerization in NF-κB proteins [4]–[6]. NFAT5 regulates the adaptation of mammalian cells to hypertonic stress by inducing the expression of osmoprotective gene products that include chaperones and a number of enzymes and transporters, such as aldose reductase (AR), whose collective function is to increase the intracellular concentration of compatible organic osmolytes that allow cells to adapt and survive in a hypertonic environment [7], [8]. In this regard, NFAT5-deficient mice suffer a progressive atrophy of their kidney medulla due to an insufficient expression of osmoprotective gene products and NFAT5-deficient cells have reduced proliferative capacity and defective expression of cyclins when cultured in hypertonic media [9], [10]–[12]. In addition, NFAT5 regulates other processes independently of osmotic stress responses, such as replication of HIV in macrophages, and migration of carcinoma or myoblast cells [13]–[16].

Activation of NFAT5 by hypertonicity involves different layers of regulation that include its rapid nuclear translocation, induction of its transcriptional activity, and its enhanced synthesis to provide additional amplification of the osmoprotective response (reviewed in [2], [17]). Previous work by us and others has shown that NFAT5 contains three major functional regions: a Rel-like DNA-binding domain (DBD) flanked by a short amino-terminal region and a large carboxy-terminal domain [5]. The *nfat5* gene encodes three isoforms, NFAT5a, b and c, which have identical amino acid composition except in their respective amino termini. The longest isoform is NFATc, with 76 additional amino acids before the first methionine of isoform NFAT5a, which is the shortest [4]. The DBD confers DNA-binding specificity and also contains the dimerization surface that makes NFAT5 a constitutive homodimer [3], [5], [6]. The carboxy-terminal domain, which is acidic and glutamine-rich, spans more than 900 amino acids and is responsible for its hypertonicity-induced transcriptional activity [5], [18]. Nucleo-cytoplasmic trafficking of NFAT5 is regulated by sequences located within its amino-terminal region, preceding the Rel-like DNA-binding domain. In isotonic conditions, NFAT5 can be found in the cytoplasm or distributed in both the cytosol and the nucleus [5], [19], whereas in response to hypertonic stress it accumulates in the nucleus, binds to regulatory regions in target genes, and activates their transcription [5], [19]. Nuclear translocation of NFAT5 in response to hypertonicity is mediated by a nuclear localization sequence (NLS) common to the three NFAT5 isoforms [20], whereas its export in the absence of hypertonic stress is regulated by two distinct motifs, a CRM1-dependent nuclear export signal (NES) found only in the long isoform NFAT5c, and a CRM1-independent, CK1-regulated, auxiliary export domain (AED) found in the three isoforms [20]–[22].

Besides stimulus-specific regulation, transcriptional processes in proliferating cells are subjected to another layer of control imposed by mitosis. It is well established that transcriptional silencing during mitosis is a process that inactivates the basal transcription machinery, specific transcription factors, and chromatin structural proteins [23], [24]. This event is not governed by a universal mechanism, but rather the exclusion of individual transcription factors from mitotic chromatin can be regulated by distinct means. Examples of transcription factors that are inactivated during mitosis are the large family of C2H2 zinc fingers and the POU family of homeodomain proteins [25]–[27]. These regulatory mechanisms could serve to inactivate specific proteins during mitosis, but can also be considered as a window in the cell cycle for resetting transcriptional programs [28]. In this regard, mitosis can provide cells with a mechanism to shut down transcriptional processes that will no longer be needed in the next cell cycle, unless the triggering stimulus still persists. In a population of cycling cells exposed to a sustained source of stress, the equilibrium between resetting and reactivating stress-responsive factors must be finely regulated.

In this work, we describe that the subcellular localization of NFAT5 and its capacity to bind to chromatin are sensitive to the passage of cells through mitosis. We report that the carboxy-terminal domain (CTD) of NFAT5 causes its exclusion from mitotic chromatin in both isotonic and hypertonic conditions. NFAT5 mutants lacking this domain bound to mitotic chromatin, which in turn caused both their constitutive nuclear localization and association with chromatin in interphase in the absence of hypertonic stimulation. This regulatory mechanism was observed in different NFAT5 isoforms. Exclusion from mitotic chromatin was mediated by different parts of the CTD of NFAT5, some of which overlapped with transactivation domains. These results reveal a novel function of the CTD of NFAT5, which, by causing its dissociation from DNA in mitosis, regulates the subcellular localization and chromatin association of this protein in interphase. Therefore, our findings indicate that the mitotic resetting of NFAT5 contributes to ensure that its nuclear localization and binding to chromatin in the next cell cycle are induced in a stimulus-specific manner.

## Results

### The carboxy-terminal domain (CTD) of NFAT5 prevents its association with mitotic chromatin

**Figure 1:**
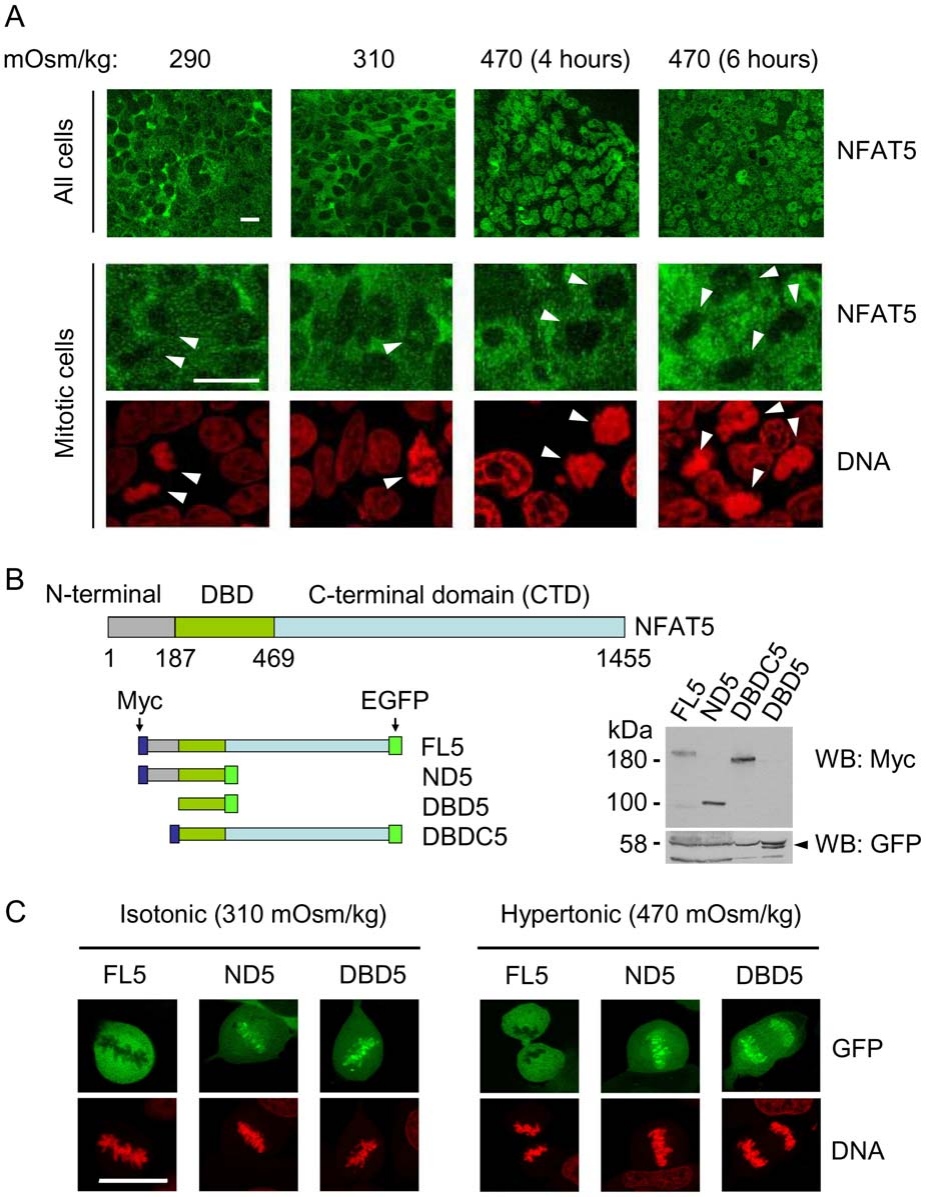
Displacement of NFAT5 from mitotic chromatin. (A) Endogenous NFAT5 was detected with an antibody specific for its DNA binding domain and visualized by immunofluorescence and confocal laser microscopy in asynchronous cultures of HEK293 cells after a fresh change of media to either isotonic conditions (290 or 310 mOsm/kg, 6 hours) or hypertonic conditions (470 mOsm/kg, 4 and 6 hours). Pictures at lower magnification show a general view of the culture, and higher magnification images display individual mitotic cells (marked by arrowheads). Scale bar is 20 µm. (B) Schematic representation of NFAT5a constructs FL5, ND5, DBD5 and DBDC5, and the position of their respective tags: FL5, ND5 and DBDC5 were tagged with 6 copies of a Myc epitope at their amino terminus and GFP at their carboxy terminus. DBD5 was tagged with GFP at its amino terminus. Constructs were expressed in HEK293 cells and detected by Western blotting with anti-Myc antibody (FL5, ND5 and DBDC5) or anti-GFP antibody (DBD5). (C) Representative confocal microscopy images of mitotic cells in asynchronous cultures of HEK293 cells expressing the indicated GFP-tagged constructs in isotonic conditions (310 mOsm/kg) or after a 6-hour exposure to hypertonic conditions (470 mOsm/kg).

**Figure 2:**
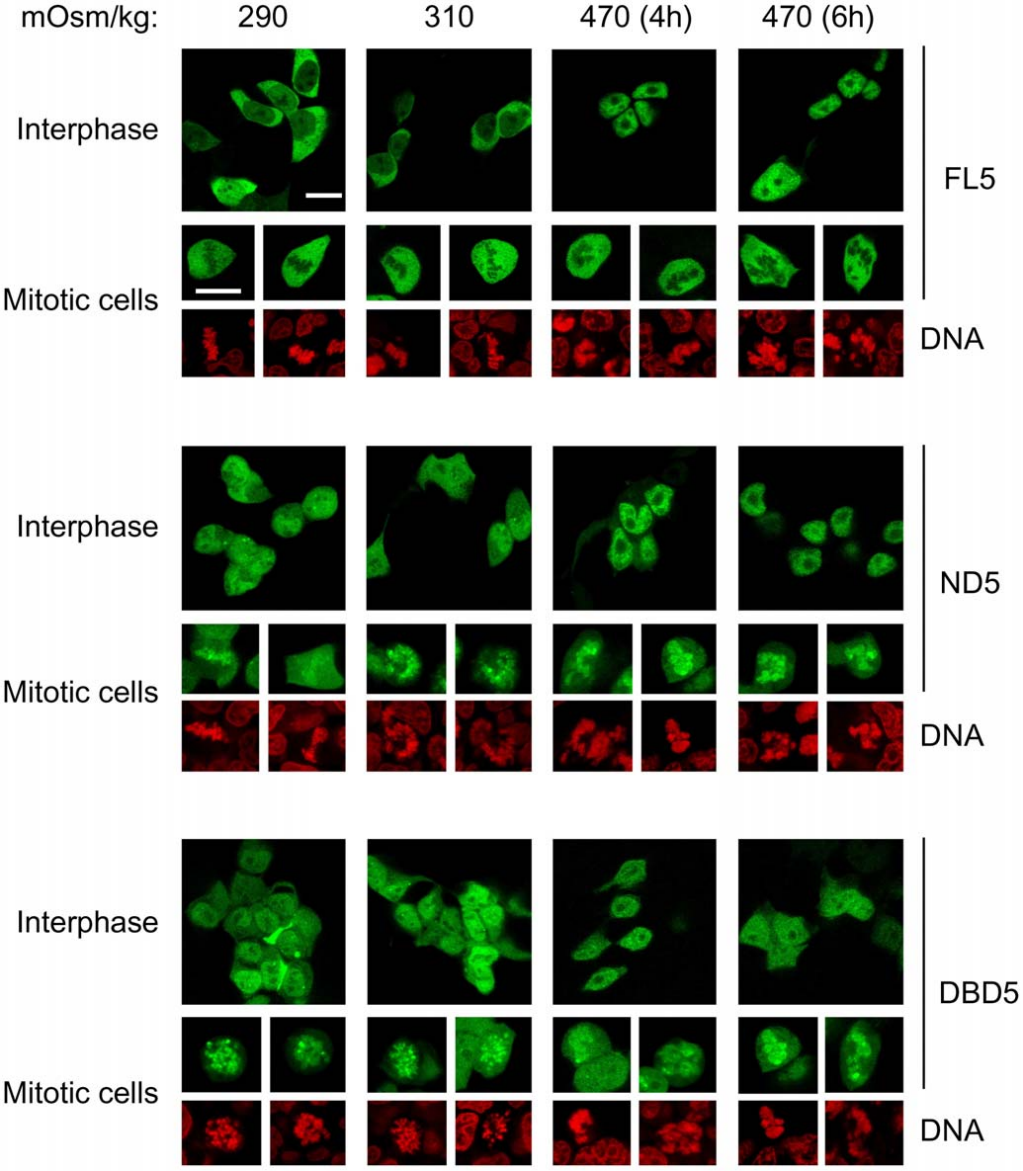
Effect of the carboxy-terminal domain (CTD) on the association of NFAT5a with mitotic chromatin and its subcellular distribution in interphase. GFP-tagged constructs FL5, ND5 or DBD5 were expressed in HEK293 cells. Their subcellular distribution in interphase and association with mitotic chromatin in isotonic (290 or 310 mOsm/kg, 6 hours) and hypertonic conditions (470 mOsm/kg, 4 and 6 hours) was analyzed by confocal microscopy. Scale bar is 20 µm. Results shown are representative of three independent experiments.

As the distribution of many chromatin-binding proteins during mitosis can be readily visualized using cell imaging techniques by their exclusion from condensed chromatin between prophase and telophase [24], our first approach consisted on analyzing whether NFAT5 was retained on, or excluded from, mitotic chromatin in cells growing in isotonic or hypertonic conditions. Confocal laser microscopy analysis of endogenous NFAT5 in HEK293 cells showed that it had a variable nucleo-cytoplasmic distribution in individual cells in asynchronous cultures in isotonic media (290–310 mOsm/kg) and accumulated in the nucleus in response to hypertonicity (470 mOsm/kg) (Figure 1A). The same experiment indicated that NFAT5 was excluded from mitotic chromatin in both isotonic and hypertonic conditions, despite that this stimulus induced a substantial nuclear accumulation of NFAT5 (Figure 1A) and, as will be shown later, its recruitment to chromatin in interphase. Next, we determined the regions of NFAT5 involved in the exclusion from mitotic chromatin (Figure 1B). GFP-tagged full-length NFAT5 (isoform a, FL5) [4] exhibited the same behaviour as the endogenous protein in HEK293 cells, being excluded from mitotic chromatin regardless of hypertonic stimulation (Figures 1C, 2 and Figure S1). However, NFAT5 constructs ND5 and DBD5, which lacked the carboxy-terminal domain (CTD), were constitutively associated with mitotic chromatin in either isotonic (290–310 mOsm/kg) or hypertonic conditions (470 mOsm/kg) (Figures 1C, 2 and Figure S1), and the CTD was sufficient to prevent the association of the DBD with mitotic chromatin (DBDC5 construct in Figure S1). The same result was observed in an independent cell line, U2OS (Figure S1). These results showed that the CTD of NFAT5 caused its exclusion from mitotic chromatin.

These experiments also revealed that NFAT5 constructs that bound to mitotic chromatin (ND5 and DBD5) were noticeably more nuclear than full-length NFAT5 in interphase in isotonic conditions (290–310 mOsm/kg) (Figure 2 and Figure S2A). This was particularly intriguing for the DBD5-GFP construct, since it lacks import signals [20], [22] and is structured as a dimer [5] whose predicted molecular weight as a GFP fusion is 118 kDa, so it would not be expected to diffuse through the nuclear pore. In view of these results we analyzed whether the binding to mitotic chromatin of NFAT5 mutants lacking the CTD could influence their nucleo-cytoplasmic distribution in interphase in the absence of hypertonic stress.

### Binding of NFAT5 mutants lacking the CTD to DNA in mitosis causes their constitutive nuclear localization and association with chromatin in interphase

**Figure 3:**
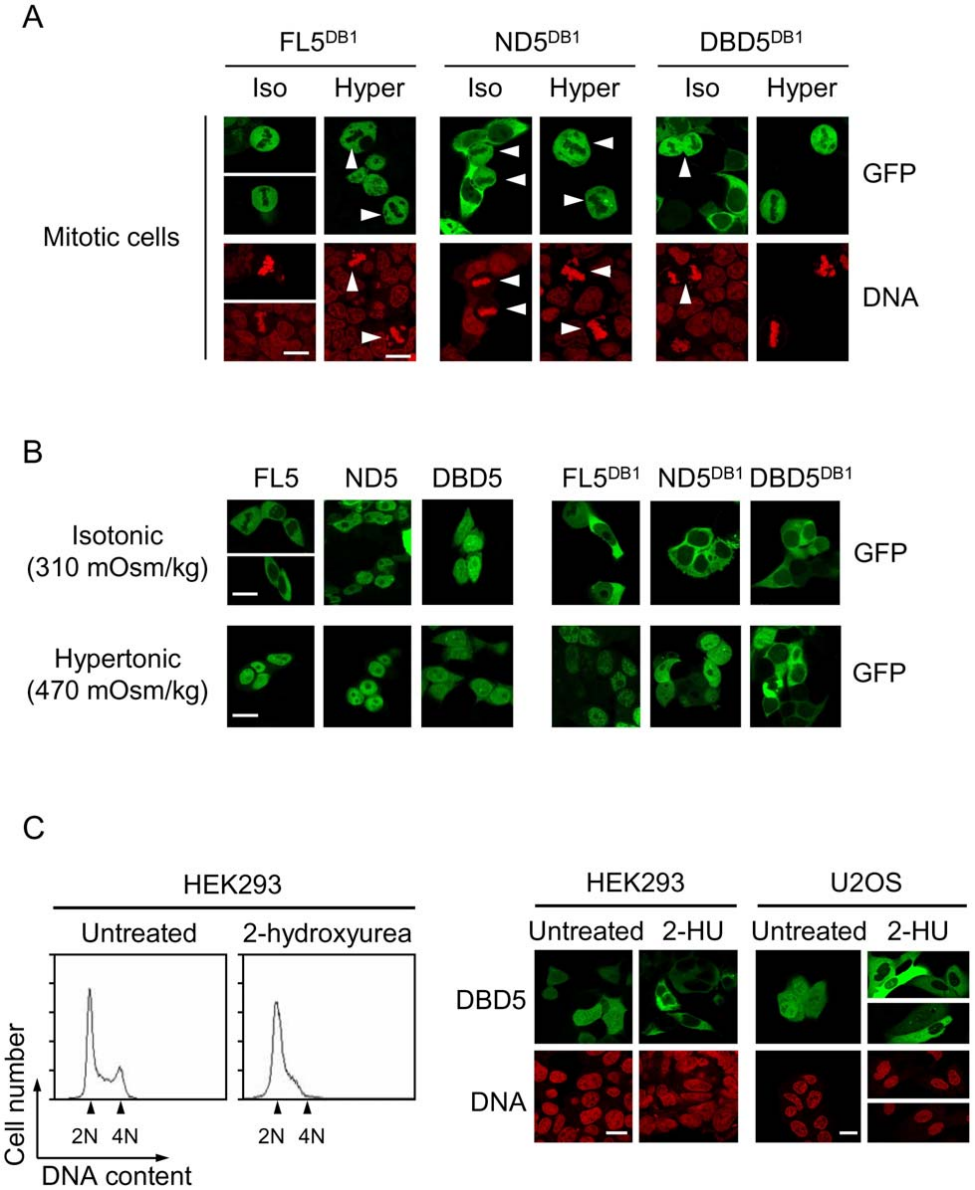
Association of the DNA binding domain of NFAT5 with mitotic chromatin is mediated by specific binding to DNA. (A) Confocal microscopy images of mitotic HEK293 cells expressing the DNA-binding mutant version (DB1) of different NFAT5a deletions tagged with GFP. Cells were grown in isotonic medium (310 mOsm/kg) or exposed to hypertonic conditions (470 mOsm/kg) during 6 hours. Scale bar is 20 µm. (B) Subcellular distribution of NFAT5a constructs capable of DNA binding (FL5, ND5, DBD5) or their respective DNA-binding mutants (FL5^DB1^, ND5^DB1^, DBD5^DB1^) in interphase HEK293 cells cultured in isotonic medium (310 mOsm/kg) or hypertonic conditions (470 mOsm/kg, 4 hours). Scale bar is 20 µm. Results are representative of four independent experiments. (C) HEK293 or U2OS cells transfected with GFP-tagged DBD5 were either left untreated or treated with 2-hydroxyurea during 48 hours to maintain them arrested in G1/S. Flow cytometry histograms show the cell cycle arrest in 2-hydroxyurea-treated HEK293 cells. Confocal microscopy images show the subcellular distribution of DBD5 in HEK293 and U2OS cells either left untreated or arrested with 2 mM 2-hydroxyurea (2-HU). Scale bar is 20 µm. The results shown are representative of four independent experiments.

**Figure 4:**
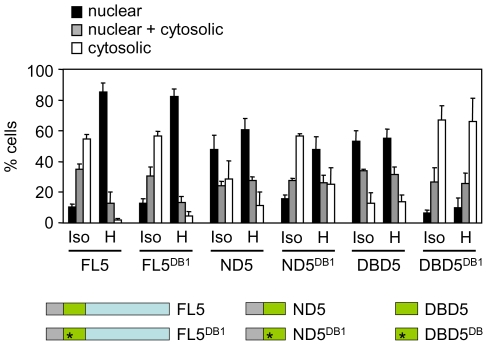
Subcellular distribution of NFAT5a constructs. Summary of subcellular localization analyses of the indicated NFAT5a constructs in interphase HEK293 cells cultured in isotonic medium (310 mOsm/kg) or exposed to hypertonic conditions (470 mOsm/kg, 4 hours). At least 150 cells with similar GFP fluorescence intensity were counted for each transfected construct in each assay. Results are the mean±SD of three independent experiments.

Mutation of the critical DNA contact residues Thr222, Glu223 and Arg226 of NFAT5a to alanine (ND5^DB1^ and DBD5^DB1^ mutants) [5] prevented the association of ND5 and DBD5 with mitotic chromatin (Figure 3A) and caused them to become cytoplasmic in interphase in isotonic conditions (Figure 3B). This result indicated that the association of ND5 and DBD5 with mitotic chromatin was due to their direct binding to specific NFAT5-recognized DNA elements. Both FL5 and ND5 DNA binding mutants (FL5^DB1^ and ND5^DB1^), which were comparably cytoplasmic in isotonic conditions, could translocate to the nucleus completely in response to hypertonicity (Figure 3B). In contrast, the subcellular localization of DBD5 was entirely dependent on its ability to bind DNA and insensitive to tonicity (see comparison of DBD5 and DBD5^DB1^ in Figures 3B and Figure S2B). Since this domain lacked import and export signals, we hypothesized that its access to DNA could occur in mitosis, and that the formation of the nuclear envelope after mitosis would keep it trapped in the nucleus in the subsequent interphase. To test this, we expressed the DBD5 in HEK293 and U2OS cells and, immediately after transfection, treated them with 2-hydroxyurea to cause their arrest at the G1/S transition so that they would not transit to mitosis (Figure 3C). Stalling of cells in G1 precluded the nuclear localization of the DBD5 in HEK293 and U2OS cells (Figure 3C), supporting the interpretation that binding to mitotic chromatin was necessary for its nuclear retention later in interphase. A summary of the results on nucleo-cytoplasmic localization of FL5, ND5, DBD5 and their corresponding DNA-binding mutants in isotonic and hypertonic conditions is shown in Figure 4. These results indicated that the constitutive nuclear localization of NFAT5 mutants lacking the CTD was due to their specific binding to DNA in mitosis.

**Figure 5:**
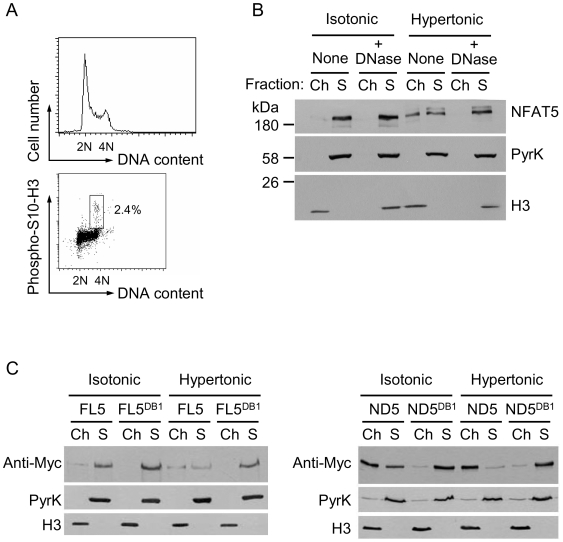
Constitutive binding to interphase chromatin of an NFAT5a mutant lacking the CTD. (A) Flow cytometry analysis of the cell cycle profile (*left histogram*) and proportion of interphase and mitotic cells (*dot plot*) in asynchronous HEK293 cultures. (B) HEK293 cells cultured in isotonic medium (310 mOsm/kg) or exposed to hypertonic conditions (470 mOsm/kg) during 4 hours were lysed and proteins were fractionated into soluble (S) or chromatin (Ch) fractions. One set of samples was treated with DNase I during lysis, which caused the release of chromatin-associated proteins to the soluble fraction. NFAT5 and markers of soluble (pyruvate kinase) and chromatin-associated proteins (histone H3) were detected by Western blotting. (C) HEK293 cells expressing Myc-tagged full length NFAT5a (FL5), a DNA-binding mutant (FL5^DB1^), or a construct comprising the amino-terminal region plus DNA-binding domain (ND5) (diagram in Figure 1B) and its DNA-binding mutant (ND5^DB1^) were cultured in isotonic medium (290 mOsm/kg) or exposed to hypertonic conditions (470 mOsm/kg) during 4 hours, lysed, and proteins were fractionated into soluble (S) or chromatin (Ch) fractions. NFAT5 constructs were detected by Western blotting with an anti-Myc antibody. Results shown are representative of four independent experiments.

Since the ND5 construct bound mitotic chromatin and was nuclear in interphase in isotonic conditions, we asked whether this protein was also constitutively bound to interphase chromatin. We used chromatin fractionation assays in asynchronously growing HEK293 cells transfected with full-length NFAT5 or ND5. The large majority (>97%) of cells in asynchronous HEK293 cultures were in interphase, as determined by simultaneous analysis of phosphorylated (S10) histone H3 (indicative of mitotic cells) and DNA content by flow cytometry (Figure 5A). We observed that only a small proportion of endogenous NFAT5 was associated with chromatin in cells growing in isotonic medium, but was recruited to chromatin upon hypertonic stimulation (Figure 5B). The fractionation was verified by the detection of pyruvate kinase only in the soluble fraction and histone H3 only in the chromatin fraction. Treatment of the lysate with DNase I before extracting the chromatin removed NFAT5 entirely from this fraction, confirming its specific association with DNA (Figure 5B). In this type of assay, transfected full-length NFAT5a (FL5) behaved as the endogenous protein, being found in the soluble fraction in isotonic conditions and bound to chromatin upon hypertonic stimulation (Figure 5C). Recruitment of NFAT5a to chromatin was eliminated by mutation of its DNA-binding residues (FL5^DB1^ and ND5^DB1^ mutants in Figure 5C), indicating that chromatin-associated NFAT5 was specifically bound to its target DNA sites. In contrast to full-length NFAT5, ND5 was already bound to chromatin in asynchronous cultures in isotonic conditions, but still could increase its association with chromatin under hypertonic stress (Figure 5C). These results indicated that the CTD caused the exclusion of NFAT5 from chromatin specifically in mitosis, but did not inhibit its binding to chromatin in response to hypertonicity in interphase.

### Contribution of the carboxy-terminal domain (CTD) and import/export signals in the amino-terminal region of NFAT5 to its subcellular localization in isotonic and hypertonic conditions

**Figure 6:**
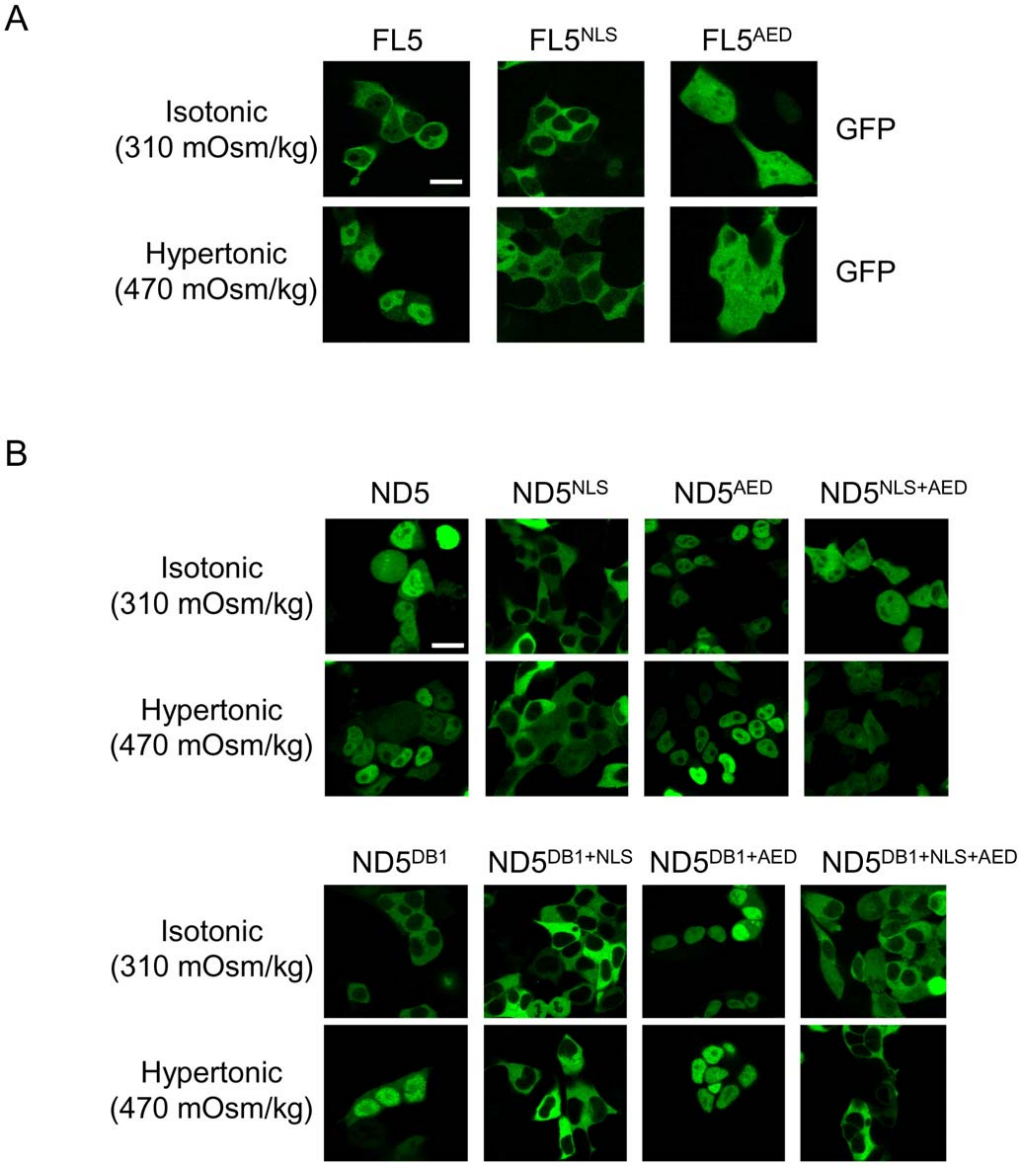
Regulation of the localization of NFAT5a by its CTD, nuclear localization signal (NLS), the auxiliary export domain (AED) and DNA binding. (A) Confocal microscopy images of HEK293 cells (whole culture or mitotic cells) expressing wild-type NFAT5a (FL5), an NLS mutant (FL5^NLS^), or an AED mutant (FL5^AED^). Cells were grown in isotonic medium (310 mOsm/kg) or exposed to hypertonic conditions (470 mOsm/kg) during 4 hours. Scale bar is 20 µm. (B) Confocal microscopy images of HEK293 cells expressing DNA-binding-competent (*upper panel*) or DNA-binding-disabled (DB1, *bottom panel*) versions of ND5 with intact NLS and AED motifs or with the NLS, AED or both mutated. Cells were grown in isotonic medium (310 mOsm/kg) or exposed to hypertonic conditions (470 mOsm/kg) during 4 hours. Scale bar is 20 µm. Results shown are representative of three independent transfections.

The results described above indicated that dissociation of NFAT5 from mitotic DNA could influence the equilibrium between nuclear import and export mechanisms in interphase. We examined the relative contribution of import, export and DNA binding to the subcellular localization of NFAT5 in isotonic conditions. These experiments, as those in previous figures, were done with isoform NFAT5a, which has a nuclear localization sequence (NLS) and an auxiliary export domain (AED), but lacks the nuclear export signal (NES) exclusive of the longer isoform NFAT5c [20]. Mutation of the NLS impaired the translocation of full length NFAT5a in response to hypertonicity, whereas removal of its AED caused it to localize in the nucleus in isotonic conditions (Figure 6A), indicating that the NLS and AED were sufficient to control the nucleo-cytoplasmic transport of NFAT5a in isotonic or hypertonic conditions. However, since full-length NFAT5a was naturally excluded from mitotic chromatin (Figures 1C and 2), we tested the effect of the NLS and AED mutations in the ND5 construct to take into account the specific contribution of mitotic DNA binding on import and export in interphase. We then observed that the NLS mutant was cytoplasmic and unable to translocate in response to hypertonicity, whereas the AED mutant was constitutively nuclear in isotonic conditions (Figure 6B). Both mutants were capable of binding to mitotic chromatin (Figure S3). The result that inactivating the NLS of ND5 suppressed its nuclear localization in isotonic conditions was intriguing, since in view of our previous results (Figures 1C and 2) we would have expected that this mutant (ND5^NLS^) should have been retained to some extent in the nucleus due to its ability to associate with chromatin during mitosis. These results suggested that the nuclear retention of ND5 in isotonic conditions was due to both its binding to chromatin during mitosis and nuclear import in interphase, and that lack of either allowed the AED to become dominant and cause its export. Consistent with this, a double mutant lacking both the NLS and AED (ND5^NLS+AED^) displayed a pancellular distribution in interphase, which was dependent only on its DNA binding capacity and was insensitive to hypertonicity (comparison between ND5^NLS+AED^ and ND5^DB1+NLS+AED^ in Figure 6B and Figure S3). These results indicated that both the NLS and AED of NFAT5a were active in isotonic conditions, and that the CTD-mediated exclusion from mitotic chromatin was required to shift its export/import balance towards a cytoplasmic localization.

**Figure 7:**
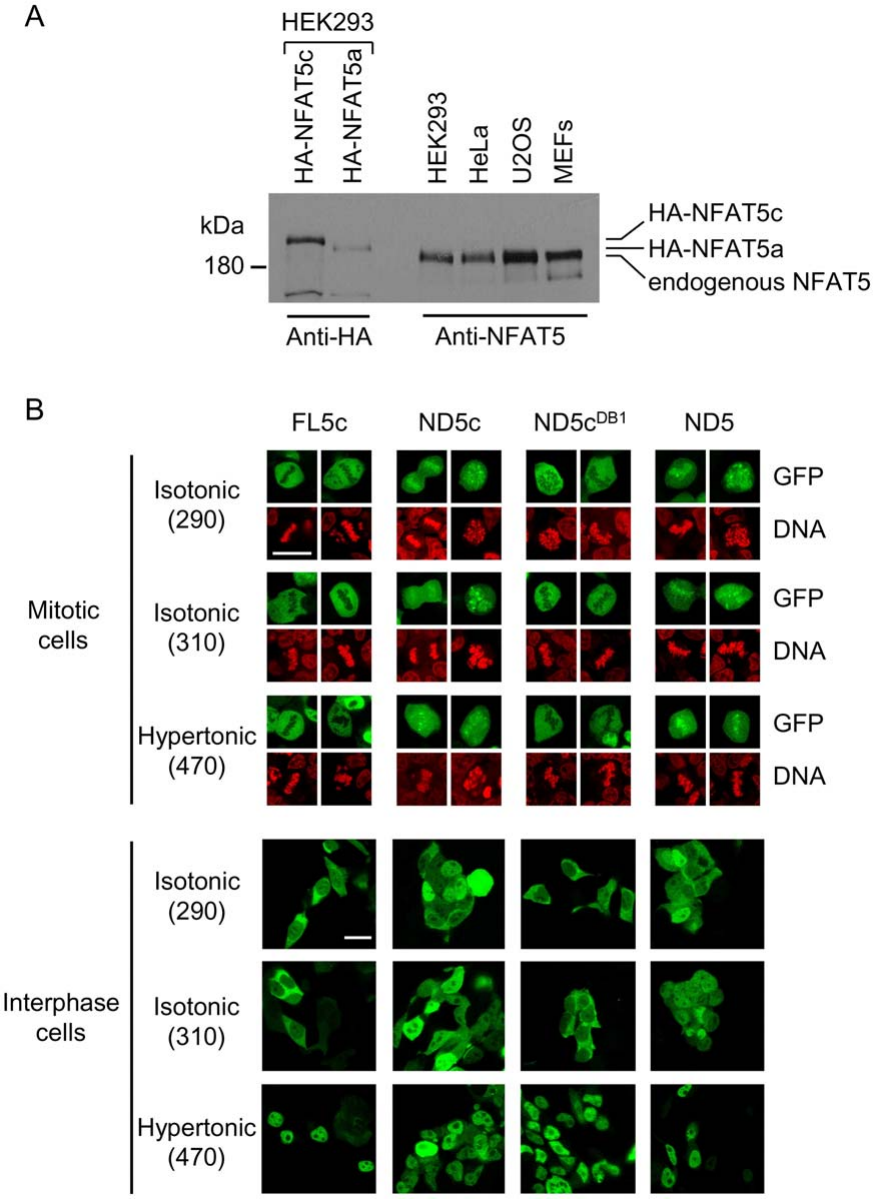
Association with mitotic chromatin and subcellular localization of an NFAT5c mutant lacking its CTD. (A) Comparison of the relative mass of endogenous NFAT5 in different cell lines. HA-tagged NFAT5c and HA-tagged NFAT5a expressed in HEK293 cells, or endogenous NFAT5 in the indicated cell lines were resolved in SDS-polyacrylamide (6%) gels. HA-tagged NFAT5c and NFAT5a constructs were detected by Western blot with the HA-specific antibody 12CA5, and endogenous NFAT5 was detected with an antibody specific for carboxy-terminal peptide conserved in all isoforms. A detailed description of this experiment is included in Materials and Methods. (B) Confocal microscopy images of mitotic and interphase HEK293 cells expressing full-length NFAT5c (FL5c), a mutant lacking its CTD (ND5c) or a mutant lacking the CTD and unable to bind DNA (ND5c^DB1^). Cells transfected with a construct of NFAT5a lacking the CTD (ND5) were analyzed in the same experiment for comparison. Cells were cultured in isotonic medium (290 and 310 mOsm/kg) or exposed to hypertonic conditions (470 mOsm/kg) during 4 hours. Scale bar is 20 µm. Results shown are representative of four independent transfections (see Figure S4).

Until here, we had used constructs corresponding to isoform NFAT5a. Three mRNA isoforms of NFAT5 have been described, all of which encode predicted proteins that are identical along 1455 amino acids and they only differ in that NFAT5b has 29 extra amino acids in its amino-terminus with respect to the shorter NFAT5a, and isoform NFAT5c is 47 amino acids longer than NFAT5b and 76 amino acids longer than NFAT5a [4]. A distinct feature of isoform NFAT5c is that it has a CRM1-dependent canonical nuclear export signal (NES) in its first 15 amino acids, which is absent from isoforms a and b [20]. At present it is unknown whether all isoforms are expressed as proteins in different cell types. To detect the potential presence of various protein isoforms we prepared lysates of several cell types and did Western blot with an antibody specific for the carboxy terminus, which is common to all three isoforms. As controls, we used lysates of HEK293 cells transfected with NFAT5a or NFAT5c constructs tagged with 3 copies of an HA epitope, which added 38 amino acids to each respective NFAT5 isoform. The anti-NFAT5 antibody detected a single band in SDS-polyacrylamide gels of lysates of HEK293, U2OS, HeLa and mouse embryo fibroblasts (MEFs) (Figure 7A). The mobility of endogenous NFAT5 in HEK293 was faster than that of the recombinant HA-NFAT5a expressed in the same cell line and clearly faster than that of HA-NFAT5c (Figure 7A). Similarly, endogenous NFAT5 in the other cell lines (U2OS, HeLa, MEFs) migrated faster than HA-NFAT5a and HA-NFAT5c. Hence, this experiment suggested that the predominant isform in them corresponded to NFAT5a. Nonetheless, we analyzed whether the long NFAT5c isoform also exhibited a CTD-dependent regulation of its subcellular localization in interphase. Full-length NFAT5c (FL5c) expressed in HEK293 cells was predominantly cytosolic in isotonic medium (290–310 mOsm/kg) and completely nuclear in hypertonic conditions (470 mOsm/kg) (Figure 7B and Figure S4). NFAT5c was also excluded from mitotic chromatin in isotonic as well as hypertonic conditions, and removal of its CTD caused it to bind mitotic chromatin (ND5c, Figure 7B and Figure S4). ND5c was more accumulated in the nucleus than full length NFAT5c or NFAT5a in isotonic conditions (290–310 mOsm/kg) (Figure 7B and Figure S4), which suggested that the NES and AED were not sufficient to ensure a complete cytoplasmic localization of an NFAT5c construct lacking the CTD. This observation was in agreement with experiments by Tong et al. using a similar NFAT5c deletion, which showed an appreciable degree of nuclear localization in isotonic medium, although it became cytoplasmic under hypotonic conditions [20]. The nuclear accumulation of ND5c in isotonic conditions was prevented by disrupting its DNA binding capacity (construct ND5c^DB1^) (Figure 7B and Figure S4). Altogether, our results suggested that the cytoplasmic localization of both NFAT5a and NFAT5c isoforms in isotonic conditions required the CTD-mediated exclusion from mitotic chromatin.

### Different regions of the carboxy-terminal domain of NFAT5 mediate its exclusion from mitotic chromatin

**Figure 8:**
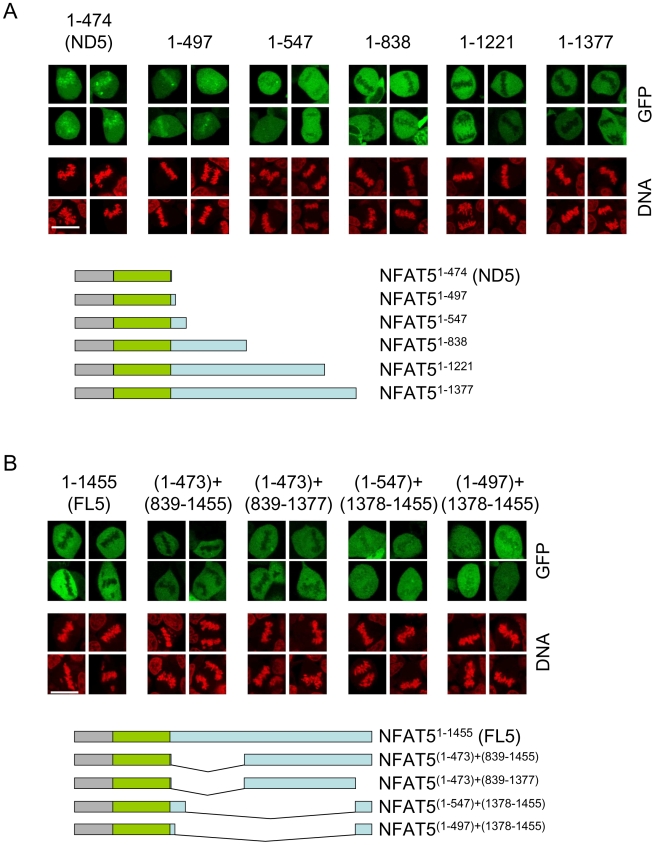
Effect of different regions of the CTD on the exclusion of NFAT5a from mitotic chromatin. Representative confocal microscopy images of mitotic cells in asynchronous cultures of HEK293 cells expressing the indicated GFP-tagged NFAT5a constructs and cultured in isotonic conditions (310 mOsm/kg). Scale bar is 20 µm. The results shown are representative of three independent experiments.

**Figure 9:**
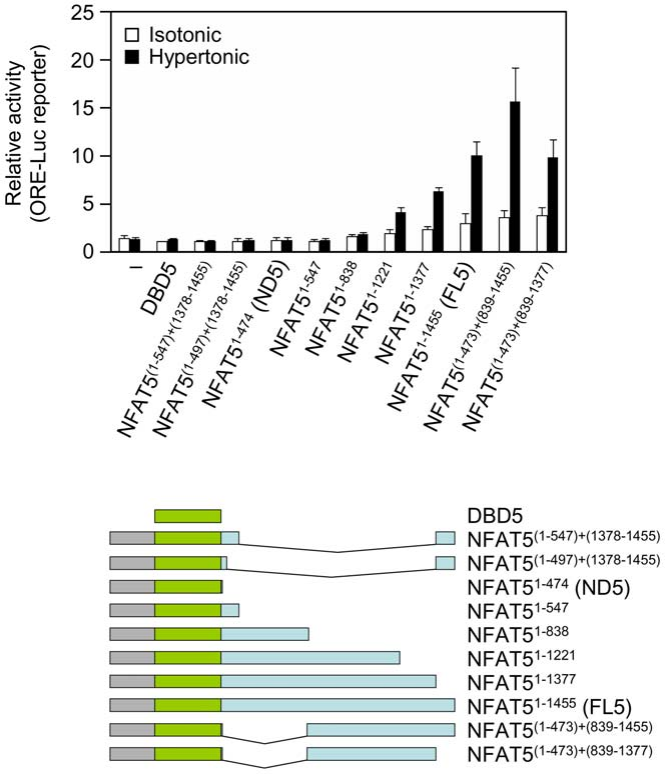
Transcriptional activity of NFAT5a CTD mutants. NFAT5-deficient MEF cotransfected with the indicated constructs and the ORE-Luc reporter were cultured in isotonic medium (310 mOsm/kg) or exposed to hypertonic conditions (510 mOsm/kg) during 20 hours. The activity of each construct is represented as relative to that of DBD5 (transcriptionally inactive) in cells cultured in isotonic medium (arbitrary value of 1). Results are the mean±SD of three independent experiments.

We next mapped which regions in the CTD of NFAT5a mediated its exclusion from mitotic chromatin (Figure 8 and Figure S5). Two regions encoded by exon 14 were independently capable of causing a strong exclusion (constructs NFAT5^1−838^ and NFAT5 ^(1−473) + (839−1377)^), whereas the regions encoded by the short exons 13 and 15 had a milder effect (constructs NFAT5^1−547^ and NFAT5 ^(1−497) + (1378−1455)^), and the region encoded by exon 12 did not displace NFAT5 from mitotic chromatin (construct NFAT5^1−497^) (Figure 8). In parallel, we analyzed the transcriptional activity of these deletion mutants in isotonic and hypertonic conditions. This analysis was done in mouse embryo fibroblasts (MEFs) devoid of NFAT5 activity [9], to prevent the contribution of endogenous NFAT5. The experiments showed that some of the regions causing the exclusion of NFAT5 from mitotic chromatin overlapped with transactivation domains, whereas others did not confer transcriptional activity (Figures 8 and 9). This is respectively illustrated by constructs NFAT5^(1−473)+(839−1377)^ and NFAT5^1−838^ (Figures 8 and 9). In addition, it was noticeable that among the mutants spanning different regions of the CTD, none of those able to bind to mitotic DNA were transcriptionally active whereas those with transcriptional activity were all excluded from mitotic chromatin. These results indicated that the CTD of NFAT5 contained two types of regions that could independently cause its exclusion from mitotic chromatin: those encoded by exons 13, 15 and part of exon 14 were not essential for its transcriptional activity, whereas the region encoded by the second half of exon 14 had a hypertonicity-activated transcriptional function in interphase that changed to repressing the binding of NFAT5 to chromatin in mitosis.

### Effect of the constitutive nuclear localization of NFAT5 on its osmoresponsive transcriptional activity

**Figure 10:**
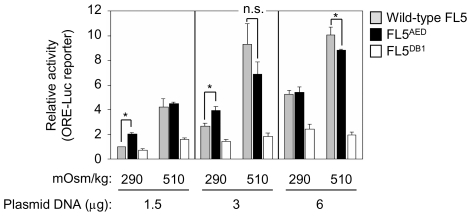
Comparison of the transcriptional activity of wild-type NFAT5a and a constitutively nuclear mutant in isotonic and hypertonic conditions. NFAT5-deficient MEF were cotransfected with a constant amount of the ORE-Luc reporter plus different concentrations of expression vectors (1.5 to 6 µg of DNA/9.4 cm^2^-well) encoding wild-type NFAT5a (FL5), a constitutively nuclear mutant (FL5^AED^) or an inactive DNA binding mutant (FL5^DB1^), and then were cultured in isotonic medium (290 mOsm/kg) or exposed to hypertonic conditions (510 mOsm/kg) during 20 hours. The activity of each construct is represented as relative to that of 1.5 µg of FL5 in isotonic medium (arbitrary value of 1). Results are the mean±SEM of four independent experiments (* p<0.05).

Our previous results raised the question of whether the control of the cytoplasmic localization of NFAT5 was necessary to prevent the activation of osmoregulatory responses in the absence of hypertonic stress. Since none of our mutants was simultaneously able to associate with mitotic chromatin and display transcriptional activity, we could not use them to address how binding to DNA in mitosis affected the transcriptional response to hypertonicity in interphase. However, since one consequence of the retention of NFAT5 mutants in mitotic chromatin was that they were localized in the nucleus in the next interphase, we used the alternative approach of testing whether a constitutively nuclear NFAT5a (export domain mutant FL5^AED^, Figure 6A) had greater basal transcriptional activity than wild-type NFAT5 in isotonic conditions. These experiments were done in MEFs lacking NFAT5 activity [9]. We found that the export mutant was a stronger activator of an osmoresponsive reporter than wild-type NFAT5 in isotonic conditions (Figure 10). This effect was observed when low amounts of NFAT5 were transfected (1.5 or 3 µg DNA/9.4 cm^2^ well), whereas overexpression of either construct produced a similarly high basal transcriptional response. These results indicated that enhancing the nuclear accumulation of NFAT5 caused a significant transcriptional response in isotonic conditions, although hypertonic stimulation was still required for full induction of its activity.

## Discussion

**Figure 11:**
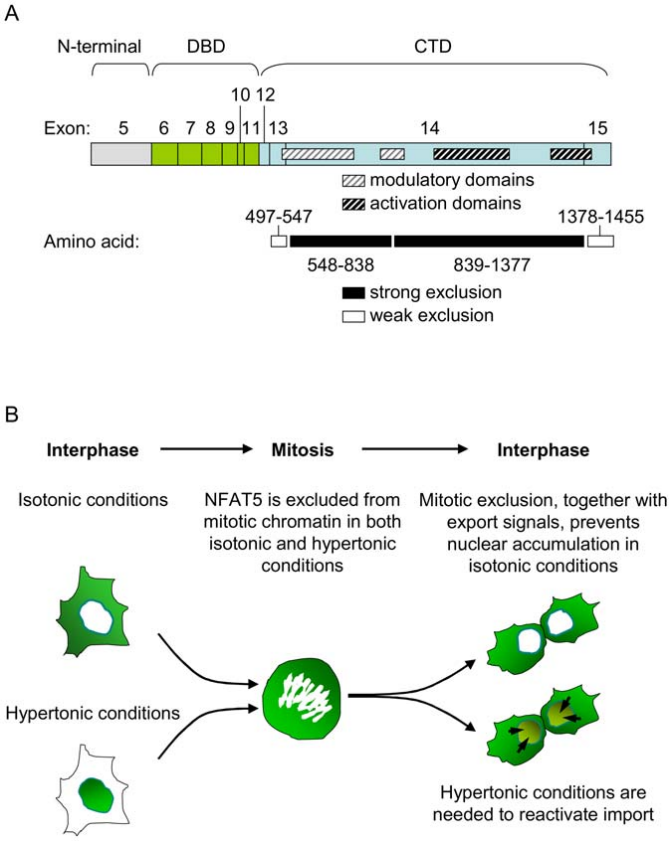
(A) Schematic representation of NFAT5a exons and functional regions in its CTD. Regions causing a strong exclusion from mitotic chromatin are indicated with black boxes and those with a weaker effect are shown in white boxes. Modulatory and activation domains characterized previously [18] are shown as hatched grey or black boxes, respectively. (B) Model illustrating the regulation of NFAT5 nucleo-cytoplasmic localization by its exclusion from mitotic chromatin. During interphase, the access of NFAT5 to target sites in chromatin is controlled by stimulus-regulated translocation across the nuclear pore. Nuclear envelope breakdown in mitosis makes DNA potentially accessible to the DBD of NFAT5. The carboxy-terminal domain inhibits the binding of NFAT5 to mitotic chromatin, contributing to localize NFAT5 in the cytosol after the nuclear envelope is formed in the next interphase. Hypertonicity reactivates translocation and association with DNA target sites. Disruption of mitotic exclusion of NFAT5 would lead to its constitutive nuclear localization and association with chromatin in the next interphase without hypertonic stimulation.

In this work we show that exclusion of NFAT5 from mitotic chromatin can reset its nucleo-cytoplasmic distribution in interphase in isotonic conditions. This process is controlled by the carboxy-terminal domain (CTD) of NFAT5, which contains several regions independently capable of preventing its association with mitotic chromatin. The overlapping of some of these regions with transactivation domains reveals that modules of NFAT5 involved in transcriptional activation in interphase can adopt a different role during mitosis, and cause the exclusion of NFAT5 from chromatin (Figure 11A). This mechanism, which functions in different NFAT5 isoforms, contributes to maintain NFAT5 in the cytosol and prevent its association with chromatin in interphase in isotonic conditions, indicating that mitosis is a significant point of control of NFAT5 (Figure 11B).

Our results reveal a previously unappreciated function of the CTD of NFAT5, and indicate that the control of its nucleo-cytoplasmic localization in cycling cells resides not only in import and export sequences located in its amino-terminal region [20], [22]. To our knowledge, this would be the first description of a transcription factor whose transactivation domain switches its function from an stimulus-specific activator of transcription in interphase to an stimulus-independent repressor of binding to DNA in mitosis, a function that serves to reset the nucleo-cytoplasmic distribution of the factor in the subsequent interphase. That exclusion of NFAT5 from mitotic DNA also occurred in hypertonic conditions indicates that binding of this factor to regulatory regions of osmoprotective genes is subject to association/dissociation cycles in proliferating cells and has to be reactivated after mitosis. This scenario raises the question of whether NFAT5-dependent osmoprotective genes use bookmarking mechanisms to facilitate their reinduction if cells exiting mitosis remain exposed to osmotic stress. Gene bookmarking has been described in the regulatory regions of genes controlled by other transcription factors that are inactivated during mitosis [28]–[30].

The relevance of NFAT5 using its carboxy-terminal domain to dissociate from mitotic chromatin is to ensure that the tonicity-sensitive import/export mechanisms may accurately control its subcellular localization and access to chromatin in the next interphase. This regulation is evident in cycling cells, since the breakdown of the nuclear envelope barrier in mitosis allows the access of NFAT5 to DNA, creating the need for preventing it from remaining bound to chromatin throughout the next interphase. Exclusion from mitotic chromatin would ensure that the subcellular localization of NFAT5 in interphase is properly controlled by nuclear import and export. It can be speculated that having various independent regulatory mechanisms might provide a finer control of its function. Although the signals regulating NFAT5 import/export have not been fully elucidated, at least one recent report [21] has shown that CK1-mediated phosphorylation of the AED is important for its export function, and it is conceivable that other regulators might affect these processes. Such regulation could be hampered if NFAT5 was persistently associated with nuclear DNA as result of having been retained in chromatin during mitosis. It is also possible that sustained activity of this factor due to uncontrolled nuclear localization might be detrimental for the cell. For instance, it has been reported that overaccumulation of organic osmolytes in cells not subjected to osmotic stress can be toxic [31]–[33], and thus limiting NFAT5 activity in the absence of stress might help to prevent such negative effects. In this regard, our comparison of a wild-type NFAT5 construct with a constitutively nuclear one suggested that nuclear localization alone without hypertonicity could cause a moderate transcriptional response. On the other hand, the significance of a complex control of NFAT5 subcellular localization in isotonic conditions might be found outside hypertonic stress responses, since this factor can also regulate osmotic stress-unrelated processes, such as integrin-induced migration, cell differentiation and viral replication [13]–[16].

The sustained association of NFAT5 with chromatin through mitosis and interphase caused by deletion of its CTD was consistent with the high stability of NFAT5-DNA complexes described previously in vitro [6]. In this regard, NFAT5 has lower affinity for DNA than the calcineurin-regulated NFATc proteins, but NFAT5-DNA complexes are more stable than NFATc-DNA ones [6]. NFAT5 and NFATc proteins can bind to the same core GGAAA element [3], [34] and, although the number of sites that could recruit either type of factor in the context of *in vivo* chromatin is probably limited, a deregulated NFAT5 that bound to DNA in mitosis and remained bound to it through interphase could hinder the access of NFATc proteins to certain sites.

Although other Rel proteins are not known to be regulated by similar mechanisms, the inhibitory effect of the carboxy-terminal domain of NFAT5 might recall the ability of the IκB-like carboxy-terminal regions of the NF-κB proteins p100 and p105 to inhibit their binding to DNA [35]–[38]. However, there is no obvious homology between the carboxy-terminal domain of NFAT5 and IκBs other than their overall acidic nature. In this regard, NFAT5 does not contain the characteristic ankyrin repeats of IκB proteins, nor it is proteolyzed during activation. In addition, these IκB-like domains prevent the activation of NF-κB in interphase in the absence of specific stimuli, whereas the carboxy-terminal domain of NFAT5 is inhibitory only in mitosis, and independently of whether NFAT5-activating conditions are present or not.

We observed an extensive overlapping of NFAT5 regions that caused its exclusion from mitotic chromatin (amino acids 547–838 and 839–1377 in NFAT5a) with those previously shown to induce its transcriptional activity (amino acids 960–1397) or enhance it (amino acids 539–876) [18] (Figure 11A). A relevant question is how these regions can switch NFAT5 from a transcriptionally active state, in which NFAT5 must be bound to chromatin, to another in which it is excluded from DNA. It can be proposed that mitosis-specific modifications of either NFAT5 or the chromatin, or a repressor protein interacting with NFAT5 in mitosis might disrupt the binding of this factor to DNA. On the other hand, the CTD of NFAT5 has a high content of serines (145 residues) and threonines (74 residues), which together comprise more than 22% of this domain, suggesting the possibility of a complex phosphorylation-dependent regulation. The observation that different parts of the CTD of NFAT5 could independently inhibit its binding to mitotic chromatin also raises the question of whether these regions share a common feature or instead each of them uses a different mechanism. However, these regions do not display obvious sequence similarities between them, nor with other proteins known to be excluded from mitotic DNA, such as the C2H2 zinc finger or the POU families of transcription factors [25]–[27]. Besides these questions, it remains to be known whether transactivation domains in other proteins can also play a dual role as activators of transcription in interphase and repressors of DNA-binding in mitosis, and whether they can reset the nucleo-cytoplasmic localization of transcription factors by controlling their association with mitotic chromatin.

## Materials and Methods

### Cell lines, hypertonicity treatment, cell cycle arrest

The human embryonic kidney cell line (HEK293), the osteosarcoma-derived U2OS cell line (American Type Culture Collection), the human cervix adenocarcinoma HeLa cell line, and NFAT5^−/−^ mouse embryonic fibroblasts (MEF) [9], were grown in Dulbecco's Modified Eagle's Medium (DMEM) (Gibco) supplemented with 10% fetal bovine serum (Gibco), 2 mM L-glutamine (Gibco), 50 µM beta-mercaptoethanol (Gibco), 1 mM sodium pyruvate (Gibco) and the antibiotics penicillin (100 units/ml) and streptomycin (100 µg/ml) (Gibco) (complete medium). This culture medium was made hypertonic, as indicated in figure legends, by addition of NaCl from an sterile 5 M stock solution. Osmolality was measured with a vapor pressure osmometer (VAPRO 5520 from Wescor). For the G1 arrest experiments, HEK293 or U2OS cells were switched to complete media with 2 mM 2-hydroxyurea (Calbiochem) immediately after transfection and cultured during 48 hours. G1 arrest was confirmed by flow cytometry analysis in cells labeled with 5 µg/ml of the DNA dye Hoechst 33342 (Sigma-Aldrich) during the last hour of culture. Cells were analyzed using a BD-LSR cytometer (Becton Dickinson) and the Cellquest software V3.3 (Becton Dickinson).

### Fluorescence microscopy

HEK293 cells transfected with different GFP-fusion constructs (described below) were cultured for 48 hours on sterile glass coverslips coated with either polylysine (Sigma-Aldrich, 0.01% w/v in water) or collagen (Sigma-Aldrich, 0.005% in 0.02 N acetic acid). Cells were fixed with 3% paraformaldehyde in 0.1 M Phosphate buffer pH 7.4, washed three times with 0.5% NP-40 in PBS, and stained with propidium iodide (Sigma-Aldrich, 30 µg/ml in 0.03% Sodium Citrate solution) for 30 min at 37°C or with 2 µM TO-PRO3 iodide (Invitrogen) for 30 min at room temperature. For U2OS cells, the process was similar but cells were plated directly on top of sterile glass coverslips. Cells were visualized by confocal laser microscopy (Leica TCS SP2) and images were acquired using the Leica Confocal Software v.2.6.1 Build 1537. Optical section thickness was fixed to 400 nm. For the detection of endogenous NFAT5, HEK293 cells were cultured on top of collagen-coated (Sigma-Aldrich) coverslips for 48 hours. After that, hypertonic stimulation was done for 4 and 6 hours, cells were fixed with 3% paraformaldehyde, and stained with a previously described rabbit polyclonal anti-NFAT5 antibody specific for its DNA binding domain [3]. DNA was stained with 2 µM TO-PRO3 iodide as indicated above, and cells were visualized by confocal laser microscopy.

### Transfection of cell lines

HEK293 cells were plated onto 35 mm (9.4 cm^2^) tissue culture dishes at 0.2×10^6^ cells in 2 ml of culture media. 20 hours after that, cells were transfected by a calcium phosphate protocol [39] using 2 to 6 µg of different NFAT5 expression vectors (as indicated) and an empty plasmid (pCMV-HA vector, Clontech) to normalize for the total amount of transfected DNA. NFAT5 ^−/−^ MEF cells were plated onto 35 mm tissue culture dishes at 0.7×10^5^ cells in 2 ml of media. 20 hours after that, cells were transfected by calcium phosphate using 450 ng of the ORE-Luc reporter plasmid [9], 600 ng of TK-Renilla plasmid (Promega), 1.25 to 2.5 µg of the different NFAT5 expression vectors and an empty plasmid (pCMV-HA plasmid) used to adjust the final amount of transfected DNA to 6 µg.

### Reporter assays

NFAT5^−/−^ MEF were transfected as indicated above. 12 hours later, transfection was stopped by renewing the complete media. 20 hours after that, cells were left untreated (310 mOsm/Kg) or stimulated with 510 mOsm/Kg for another 20 hours. After that, cells were lysed using Passive Lysis Buffer (Promega) and the Luciferase and Renilla activities were measured using the Dual-luciferase reporter system (Promega) with a Berthold FB12 luminometer (Berthold). Luciferase activity was normalized to Renilla.

### Cell lysis, chromatin fractionation, immunoblot assays and antibodies

To isolate soluble and chromatin fractions, HEK293 cells were resuspended (6×10^6^ cells/ml) in buffer A (10 mM HEPES, pH 7.9, 10 mM KCl, 1.5 mM MgCl_2_, 0.34 M sucrose, 10% glycerol, 1 mM iodoacetamide, 5 mg/ml leupeptin, 5 mg/ml aprotinin, 1 mg/ml pepstatin A, 10 mM beta-glycerophosphate, 1 mM PMSF, 10 mM NaF, 10 mM sodium orthovanadate) (as described in [40]). Next, Triton X-100 (0.1%) was added, and cells were incubated for 15 minutes on ice. Nuclei were collected by low-speed centrifugation (at 1,300 g, 4°C for 4 min), and the supernatant was separated for protein quantification and boiled in reducing 1x Laemmli buffer for 10 min at 100°C. Nuclei were washed twice in buffer A, and then were either boiled in 1x Laemmli buffer for 1 hour min at 100°C (Figure 5B), or incubated in buffer A plus DNase I (Worthington, 0.2 mg/ml, 25°C, 15 min) to extract chromatin-associated proteins (Figure 5C). Samples containing proteins released from chromatin were centrifuged (13,000 g, 4°C, 10 min) to sediment debris and the supernatants were boiled for 10 min at 100°C in 1x Laemmli reducing buffer. Whole cell extracts were done using buffer A plus 0.1% Triton X-100 supplemented with DNase I (Worthington, 0.2 mg/ml). For Western blotting, lysates were subjected to SDS-polyacrylamide gel electrophoresis and transferred to PROTRAN (BA-83, Schleider & Schuell) membranes in 25 mM Tris, pH 8.4, 192 mM glycine and 20% methanol. After blocking the membranes with 5% dry milk in 1xTBS, the following antibodies were used: rabbit polyclonal NFAT5-specific antibody raised against a carboxy-terminal peptide (Affinity Bioreagents), mouse monoclonal antibody to Myc (9E10), mouse monoclonal antibody to GFP (Covance), goat antibody to pyruvate kinase (Chemicon) or rabbit polyclonal antibody to histone H3 (Abcam). The antibody to rabbit IgG coupled to HRP was from Promega, the antibody to mouse IgG coupled to HRP was from Amershan, and the antibody to goat IgG coupled to HRP was from DAKO. Protein bands were visualized by enhanced chemiluminescence, using ECL Western Blotting Detection Reagent (Amershan).

### Intracellular detection of phosphorylated Ser10 of Histone H3

Asynchronously growing HEK293 cells were labelled following an intracellular staining protocol previously described [41]. Briefly, cells were fixed in 1.5% paraformaldehyde (Sigma-Aldrich) in PBS on ice for 15 minutes, and permeabilized with 70% ethanol at −20°C for at least 2 hours. Ethanol was removed by centrifugation and two washes with BTP Buffer (1% BSA, 0.2% Triton-X, in 1X PBS), and cells were incubated with rabbit polyclonal anti-phospho-histone H3 (Upstate-Millipore) (1 µg/10^6^ cells) for 2 hours. Bound primary antibodies were detected by incubating the cells with PE-labelled secondary antibodies (Sigma-Aldrich) for 1 hour at room temperature. DNA was then stained using 5 µg/ml Hoechst 33342 for 30 minutes at room temperature.

### Constructs

Full-length human NFAT5a tagged with 6 copies of a Myc epitope (Met-Glu-Gln-Lys-Leu-Ile-Ser-Glu-Glu-Asp-Leu-Asn-Glu) in its amino-terminal and the enhanced green fluorescence protein (GFP) at its carboxy-terminal (Myc-NFAT5-GFP); a truncated NFAT5a comprising the amino-terminal and the DNA-binding domain tagged with 6 copies of the Myc epitope in its amino-terminus and the enhanced green fluorescence protein (GFP) at its carboxy-terminus (Myc-ND5-GFP); the DNA-binding domain of NFAT5a (DBD5) tagged with an amino terminal GFP and a mutant DBD5 with T222A, E223A and R226A substitutions that cannot bind DNA (DBD5^DB1^), have been described elsewhere [3], [5]. The full length NFAT5a construct tagged with 3 copies of an hemagglutinin (HA) epitope in its amino terminus has been described [5]. A detailed description of the respective mutagenesis and subcloning steps follows. Unless specified otherwise, the template used was the NFAT5a expression vector Myc-NFAT5-GFP described in [3], corresponding to human NFAT5a (GenBank accession number: AF134870), and all the constructs had an amino-terminal tag with 6 copies of a Myc epitope and a carboxy-terminal GFP tag. All constructs newly generated for this study were verified by sequencing.

Mutants NFAT5^1−547^, NFAT5^1−838^, NFAT5^1−1221^, and NFAT5^1−1377^:

NFAT5^1−547^: PCR with primers 5′ TGG AAG ATC TCA TGA TGT TCA ACC ATT CAC 3′ and 5′ ACA CCG GTC CCT TAA AAA TAG TGG ACG ATC TTT TTT CTGC 3′ was done to amplifying a region from nucleotides 1362 to 1641 in the cDNA of human NFAT5a. The PCR product and the NFAT5 expression vector (Myc-NFAT5-GFP) were digested with BglII and AgeI restriction enzymes. The region amplified was subcloned into the expression vector. The resulting construct comprises the amino-terminal and DNA-binding domains plus a fragment of the carboxy-terminal domain comprising amino acids 473 to 547. A silent point mutation at position 1626 in the cDNA of NFAT5 was introduced to disrupt a second restriction site for BglII.

NFAT5^1−838^: PCR with primers 5′ CAG TCT AGA GAG ATA TTA CAG TCA GATG 3′ and 5′ ACA CCG GTC CCT CTG ACT GAA TCT GGG CAG 3′ was done to amplifying a region from nucleotides 1924 to 2514 in the cDNA of human NFAT5a. The PCR product and the NFAT5 expression vector (Myc-NFAT5-GFP) were digested with XbaI and AgeI restriction enzymes. The region amplified was subcloned into the expression vector. The resulting construct comprises the amino-terminal and DNA-binding domains plus a fragment of the carboxy-terminal domain comprising amino acids 473 to 838.

NFAT5^1−1221^: PCR with primers 5′ CAG TCT AGA GAG ATA TTA CAG TCA GATG 3′ and 5′ ACA CCG GTC CCA TGT TTG GTG GTG GTT GCT 3′ was done to amplifying a region from nucleotides 1924 to 3639 in the cDNA of human NFAT5. The PCR product and the NFAT5a expression vector (Myc-NFAT5-GFP) were digested with XbaI and AgeI restriction enzymes. The region amplified was subcloned into the expression vector. The resulting construct comprises the amino-terminal and DNA-binding domains plus a fragment of the carboxy-terminal domain comprising amino acids 473 to 1221.

NFAT5^1−1377^: PCR with primers 5′ CAG TCT AGA GAG ATA TTA CAG TCA GATG 3′ and 5′ ACA CCG GTC CAT TTT GAA TGC CAA ATA AGA 3′ was done to amplifying a region from nucleotides 1924 to 4131 in the cDNA of human NFAT5a. The PCR product and the NFAT5 expression vector (Myc-NFAT5-GFP) were digested with XbaI and AgeI restriction enzymes. The region amplified was subcloned into the expression vector. The resulting construct comprises the amino-terminal and DNA-binding domains plus a fragment of the carboxy-terminal domain comprising amino acids 473 to 1377.

Mutants NFAT5^1−497^, NFAT5^(1−547)+(1378−1455)^, NFAT5^(1−497)+(1378−1455)^, NFAT5^(1−473)+(839−1377)^, NFAT5^(1−473)+(839−1455)^, DBDC5, ND5^DB1^, FL5^DB1^: These mutants correspond to deletion or substitutions. They were done using the QuickChange XL Site-directed mutagenesis system (Stratagene).

NFAT5^1−497^ was done using the NFAT5^1−547^ expression vector as a template and the set of primers 5′ GAA GAG GCC ATG AAA GCA GGC GCC ATG GTG AGC AAG GGC 3′ and 5′ GCC CTT GCT CAC CAT GGC GCC TGC TTT CAT GGC CTC TTC 3′. The resulting construct comprises the amino-terminal and DNA-binding domains plus a fragment of the carboxy-terminal domain comprising amino acids 473 to 497.

NFAT5^(1−547)+(1378−1455)^ was done using the Myc-NFAT5-GFP expression vector as a template and the set of primers 5′ CTT CCA CTA TTT TTA AGG GCG AAG GTA ACT GTA GTC AGC TT 3′ and 5′ AAG CTG ACT ACA GTT ACC TTC GCC CTT AAA AAT AGT GGA AG 3′. The resulting construct comprises the amino-terminal and DNA-binding domains plus a fragment of the carboxy-terminal domain comprising amino acids 473 to 547 and amino acids 1378 to 1455.

NFAT5^(1−497)+(1378−1455)^ was done using the Myc-NFAT5-GFP expression vector as a template and the set of primers 5′ AGA GGC CAT GAA AGG CGA AGG TAA CTG TAG TCA G 3′ and 5′ CTG ACT ACA GTT ACC TTC GCC TTT CAT GGC CTC T 3′. The resulting construct comprises the amino-terminal and DNA-binding domains plus a fragment of the carboxy-terminal domain comprising amino acids 473 to 497 and amino acids 1378 to 1455.

NFAT5^(1−473)+(839−1377)^ was done using the NFAT5^1−1377^ expression vector (described above) as a template and the set of primers 5′ CAG ACC CAG CAG CAG CTG GTT TAT TCC CTT CAA CTG CTT C 3′ and 5′ GAA GCA GTT GAA GGG AAT AAA CCA GCT GCT GCT GGG TCT G 3′. The resulting construct comprises the amino-terminal and DNA-binding domains plus a fragment of the carboxy-terminal domain comprising amino acids 839 to 1377.

NFAT5^(1−473)+(839−1455)^ was done using the Myc-NFAT5-GFP expression vector as a template and the set of primers 5′ CAG ACC CAG CAG CAG CTG GTT TAT TCC CTT CAA CTG CTT C 3′ and 5′ GAA GCA GTT GAA GGG AAT AAA CCA GCT GCT GCT GGG TCT G 3′. The resulting construct comprises the amino-terminal and DNA-binding domains plus a fragment of the carboxy-terminal domain comprising amino acids 839 to 1455.

FL5^DB1^ and ND5^DB1^. Alanine substitutions in positions T222, E223, and R226 were introduced using the Myc-NFAT5-GFP or Myc-ND5-GFP expression vectors as a templates, respectively, and the set of primers 5′ GAG CTC GGT ACC TGG CTG CGG GCA GCG CTG GGT CAG TGA AAG 3′ and 5′ CTT TCA CTG ACC CAG CGC TGC CCG CAG CCA GGT ACC GAG CTC 3′.

DBDC5 was done using the Myc-NFAT5-GFP expression vector as a template and the set of primers 5′ GGC CCC ATG GGC GGT AAT GGA ACA TTG GAA AAC C 3′ and 5′ GGT TTT CCA ATG TTC CAT TAC CGC CCA TGG GGC C 3′. The resulting construct comprises the DNA-binding- and carboxy-terminal domains (amino acids 175 to 1455).

Myc-FL5^NLS^-GFP and Myc-ND5^NLS^-GFP were done using Myc-full length NFAT5-GFP or Myc-ND5-GFP, respectively, as templates, and point mutations were introduced to change amino acids RKR (positions 126 to 128 in NFAT5a) to AAA, as described [20]. The primers used were 5′ CCT CGT AAA TCA GCT GCA GCA AAT CCA AAG CAG3′ and 5′ CTG CTT TGG ATT TGC TGC AGC TGA TTT ACG AGG 3′.

Myc-FL5^AED^-GFP and Myc-ND5^AED^-GFP were done using the Myc-full length NFAT5-GFP or Myc-ND5-GFP, respectively, as templates and the set of primers 5′ CAG TAA CAC AGT TCA GCA GAT GTC CTG CCA GGA TGA G 3′ and 5′ CTC ATC CTG GCA GGA CAT CTG CTG AAC TGT GTT ACT G 3′. An internal deletion was done (from amino acids 56 to 80 in NFAT5a), as described [20]. The same strategy was used to generate the double mutant NLS plus AED in FL5 or ND5 using the FL5^NLS^ and ND5^NLS^ mutants as templates.

NFAT5c isoform constructs. A cDNA vector for human NFAT5 isoform c (NCBI reference NP_006590.1) was kindly provided by Osamu Ohara (Kazusa DNA Research Institute, Japan) [42]. NFAT5c was subcloned in the pEGFP-N1 vector and tagged with 3 copies of an hemagglutinin (HA) epitope in its amino terminus. Two versions of this construct were generated, one with a GFP tag in frame at its carboxy terminus (HA-NFAT5c-GFP) and another without GFP (HA-NFAT5c). The ND5c construct contained the amino-terminal and DNA binding domains of NFAT5c and was generated by subcloning to replace the Myc epitopes and amino-terminal region of Myc-ND5-GFP with a fragment encoding the HA epitopes and the amino-terminal region from HA-NFAT5c. The resulting NFAT5c construct had 3 copies of an HA epitope in its amino terminus and one GFP in frame after its DNA binding domain. The same subcloning strategy was used to generate ND5c^DB1^ in the Myc-ND5^DB1^-GFP backbone.

### Comparison of the relative mass of recombinant NFAT5a and NFAT5c isoforms with endogenous NFAT5

Lysates of HEK293 cells transfected with vectors encoding either NFAT5a or NFAT5c tagged with 3 copies of an HA epitope in their amino termini were run in the same SDS-polyacrylamide (6%) gel in parallel with lysates of several untransfected human cell lines (HEK293, U2OS and HeLa) or mouse embryonic fibroblasts (MEFs). In a first approach, gels were transferred to nitrocellulose membranes and probed sequentially with an anti-HA mouse monoclonal antibody (12CA5) followed by a rabbit antibody (anti-NFAT5) specific for a carboxy-terminal peptide common to all NFAT5 isoforms (Affinity Bioreagents). However, the anti-NFAT5, which detected endogenous NFAT5 also in the transfected HEK293 samples, gave a much stronger signal than the anti-HA antibody and obscured the detection of the band corresponding to the transfected HA-tagged constructs in the same lanes. This problem occurred regardless of the order in which antibodies were used for Western blotting. To solve this, we added a lane with molecular weight markers between the samples of transfected and untransfected cells and used it as a guide to slice the membrane along the vertical axis. Lysates from transfected HEK293 cells were used for HA detection and contained an amount of lysate equivalent to 0.36×10^6^ cells/lane, whereas lysates from untransfected cell lines for detection of endogenous NFAT5 had 0.18×10^6^ cells/lane. Both halves of the membrane were probed separately with anti-HA or anti-NFAT5 antibodies respectively and, before developing them by ECL, were realigned following the markers of the sliced lane.

### Statistical Analysis

Mean, standard deviation (SD), standard error of the mean (SEM) and statistical significance (t-Student test) were calculated using Microsoft Excel software.

## Supporting Information

Figure S1 Association of NFAT5a mutants with mitotic chromatin. Confocal microscopy images of mitotic HEK293 and U2OS cells expressing the indicated GFP-tagged constructs in isotonic conditions (310 mOsm/kg) or after a 6-hour exposure to hypertonic conditions (470 mOsm/kg). Scale bar is 20 Âµm.
(4.33 MB TIF)

Figure S2 Effect of the CTD and DNA binding on the subcellular distribution of NFAT5a constructs in interphase. (A) GFP-tagged constructs FL5, ND5 or DBD5 were expressed in U2OS cells. Their subcellular distribution in interphase in isotonic (310 mOsm/kg) and hypertonic conditions (470 mOsm/kg, 4 hours) was analyzed by confocal microscopy. Scale bar is 20 µm. Images are representative of three independent experiments. (B) HEK293 cells (left panel) or U2OS cells (right panel) expressing GFP-tagged NFAT5 DNA-binding domain (DBD5) or its DNA-binding mutant DBD5DB1 were cultured in isotonic medium (310 mOsm/kg) or exposed to hypertonic conditions (470 mOsm/kg) during 6 hours, then fixed and analyzed by confocal microscopy. Scale bar is 20 µm. The results shown are representative of four independent experiments.
(2.34 MB TIF)

Figure S3 Effect of the nuclear localization signal (NLS) and the auxiliary export domain (AED) on the association with mitotic chromatin of an NFAT5a mutant lacking its CTD. Association of the indicated ND5 constructs with mitotic chromatin in isotonic conditions (310 mOsm/kg). Scale bar is 20 Âµm. Results shown are representative of three independent transfections.
(2.52 MB TIF)

Figure S4 Comparison of the subcellular localization of NFAT5a and NFAT5c CTD deletion mutants. Summary of subcellular localization analyses of the indicated NFAT5 constructs in interphase HEK293 cells cultured in isotonic medium (290 and 310 mOsm/kg) or exposed to hypertonic conditions (470 mOsm/kg, 4 hours). At least 50 cells with similar GFP fluorescence intensity were counted for each transfected construct in each transfection. Results are the mean±SEM of four independent experiments.
(0.32 MB TIF)

Figure S5 Schematic representation of NFAT5a constructs corresponding to deletions in the CTD. Constructs were tagged with 6 copies of a Myc epitope at their amino terminus and GFP at their carboxy terminus. Western blots were done with anti-Myc antibody in lysates from HEK293 cells transfected with the indicated constructs.
(1.12 MB TIF)
