# Supplementary figures and images for: Exclusion of NFAT5 from Mitotic Chromatin Resets Its Nucleo-Cytoplasmic Distribution in Interphase
Source: PLoS One. 2009 Sep 14;4(9):e7036. doi: 10.1371/journal.pone.0007036 (PMC2737149; doi:10.1371/journal.pone.0007036)

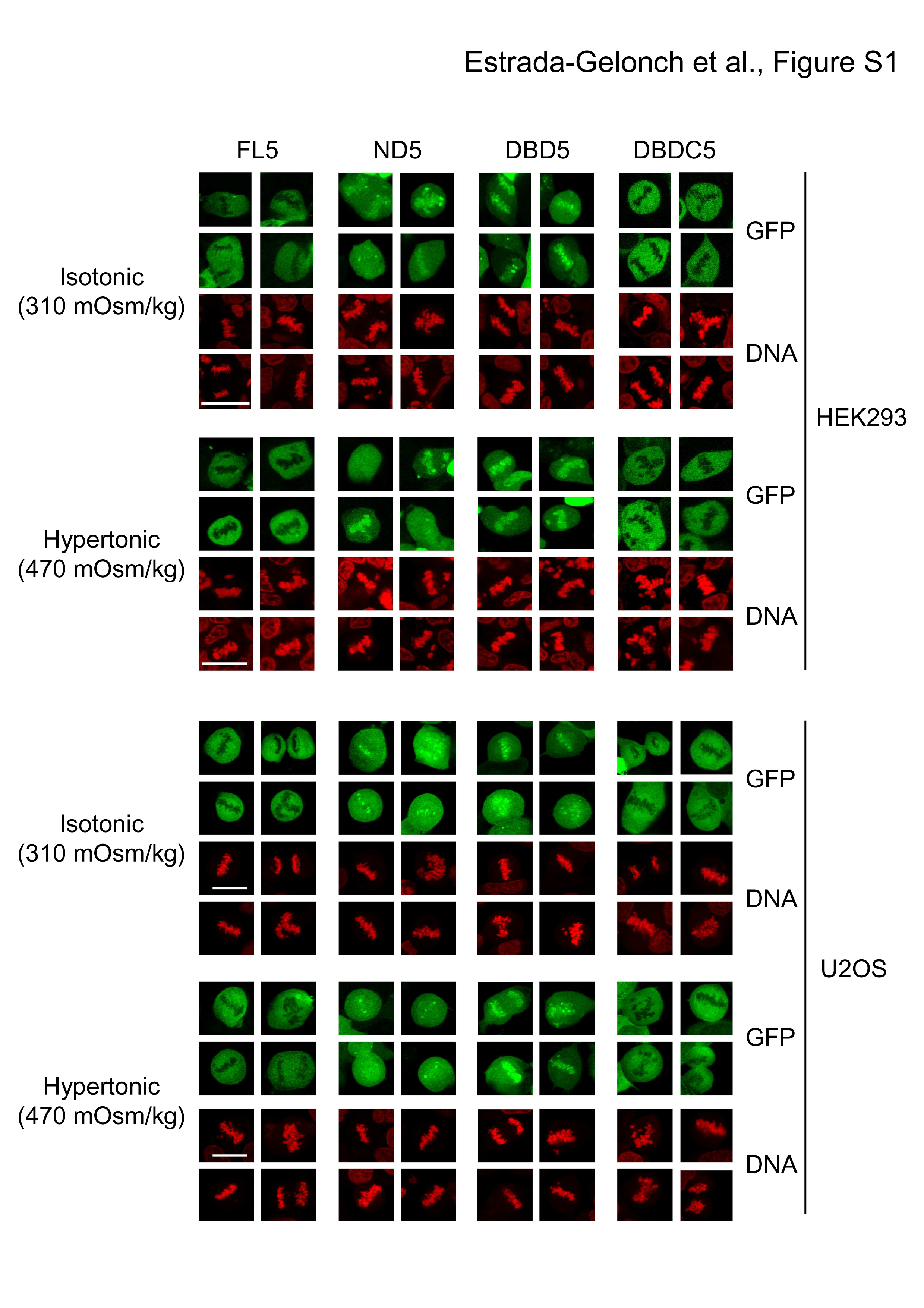

Supplement: Figure S1 — Association of NFAT5a mutants with mitotic chromatin. Confocal microscopy images of mitotic HEK293 and U2OS cells expressing the indicated GFP-tagged constructs in isotonic conditions (310 mOsm/kg) or after a 6-hour exposure to hypertonic conditions (470 mOsm/kg). Scale bar is 20 Âµm. (4.33 MB TIF) [file pone.0007036.s001.tif]

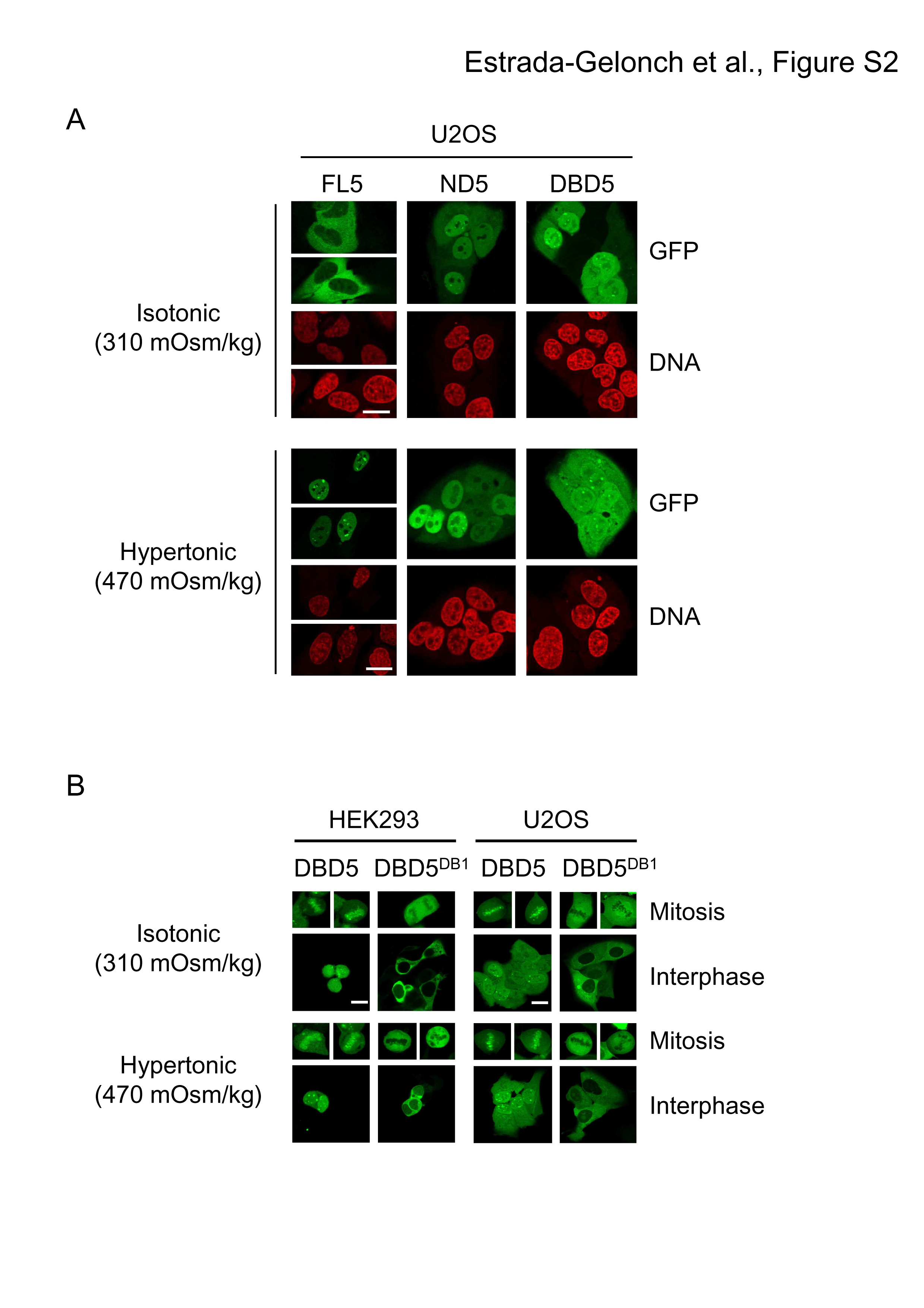

Supplement: Figure S2 — Effect of the CTD and DNA binding on the subcellular distribution of NFAT5a constructs in interphase. (A) GFP-tagged constructs FL5, ND5 or DBD5 were expressed in U2OS cells. Their subcellular distribution in interphase in isotonic (310 mOsm/kg) and hypertonic conditions (470 mOsm/kg, 4 hours) was analyzed by confocal microscopy. Scale bar is 20 µm. Images are representative of three independent experiments. (B) HEK293 cells (left panel) or U2OS cells (right panel) expressing GFP-tagged NFAT5 DNA-binding domain (DBD5) or its DNA-binding mutant DBD5DB1 were cultured in isotonic medium (310 mOsm/kg) or exposed to hypertonic conditions (470 mOsm/kg) during 6 hours, then fixed and analyzed by confocal microscopy. Scale bar is 20 µm. The results shown are representative of four independent experiments. (2.34 MB TIF) [file pone.0007036.s002.tif]

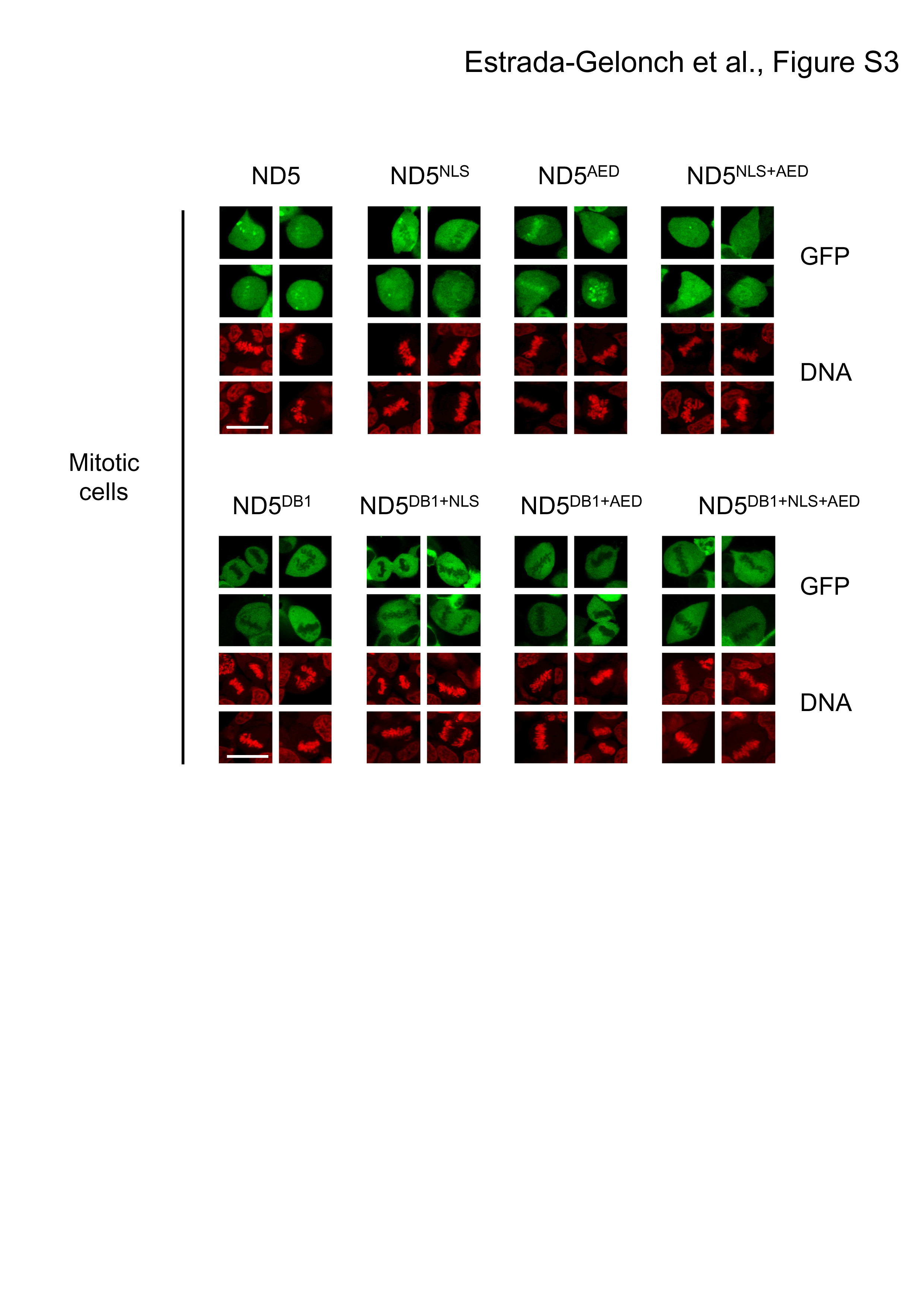

Supplement: Figure S3 — Effect of the nuclear localization signal (NLS) and the auxiliary export domain (AED) on the association with mitotic chromatin of an NFAT5a mutant lacking its CTD. Association of the indicated ND5 constructs with mitotic chromatin in isotonic conditions (310 mOsm/kg). Scale bar is 20 Âµm. Results shown are representative of three independent transfections. (2.52 MB TIF) [file pone.0007036.s003.tif]

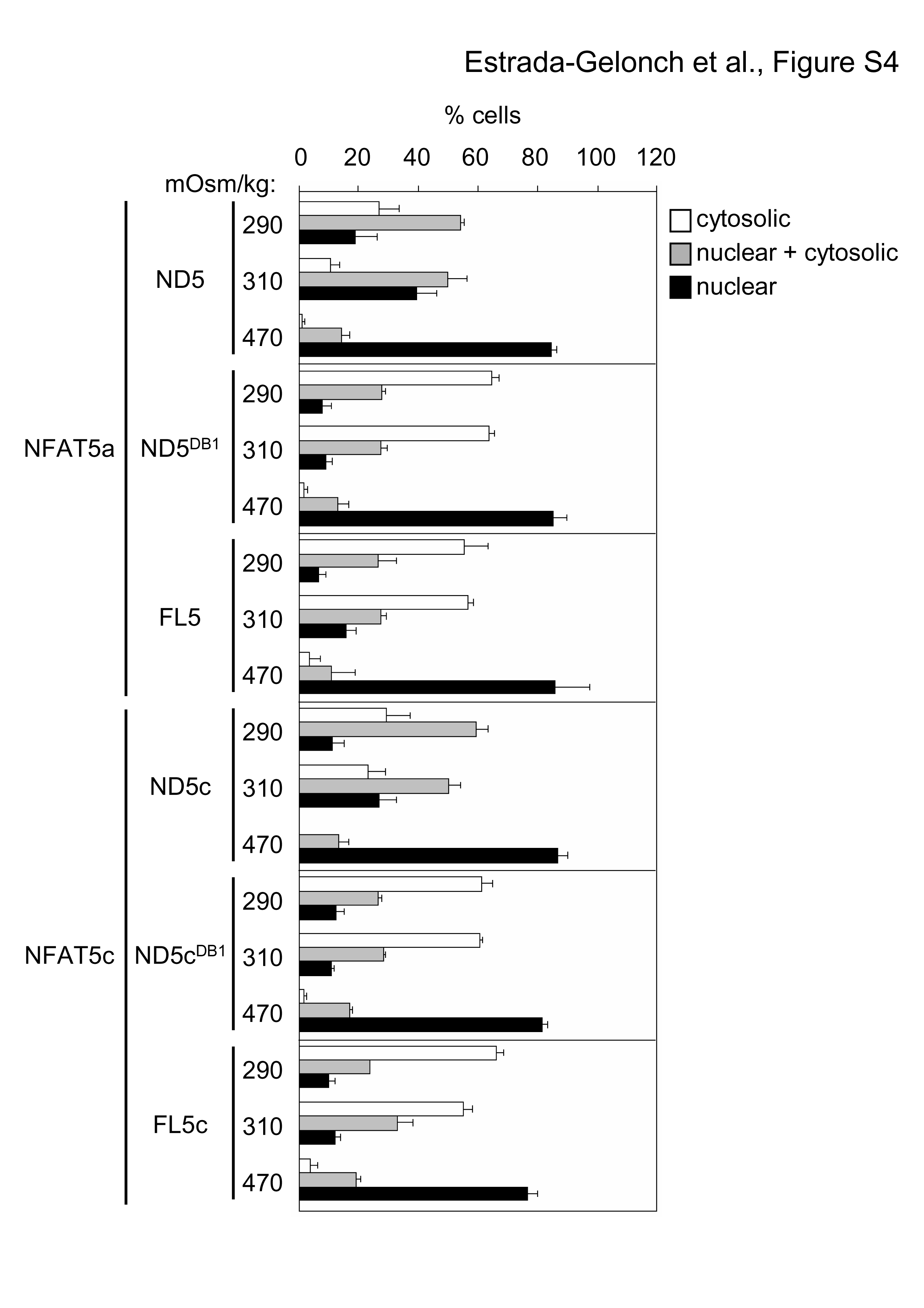

Supplement: Figure S4 — Comparison of the subcellular localization of NFAT5a and NFAT5c CTD deletion mutants. Summary of subcellular localization analyses of the indicated NFAT5 constructs in interphase HEK293 cells cultured in isotonic medium (290 and 310 mOsm/kg) or exposed to hypertonic conditions (470 mOsm/kg, 4 hours). At least 50 cells with similar GFP fluorescence intensity were counted for each transfected construct in each transfection. Results are the mean±SEM of four independent experiments. (0.32 MB TIF) [file pone.0007036.s004.tif]

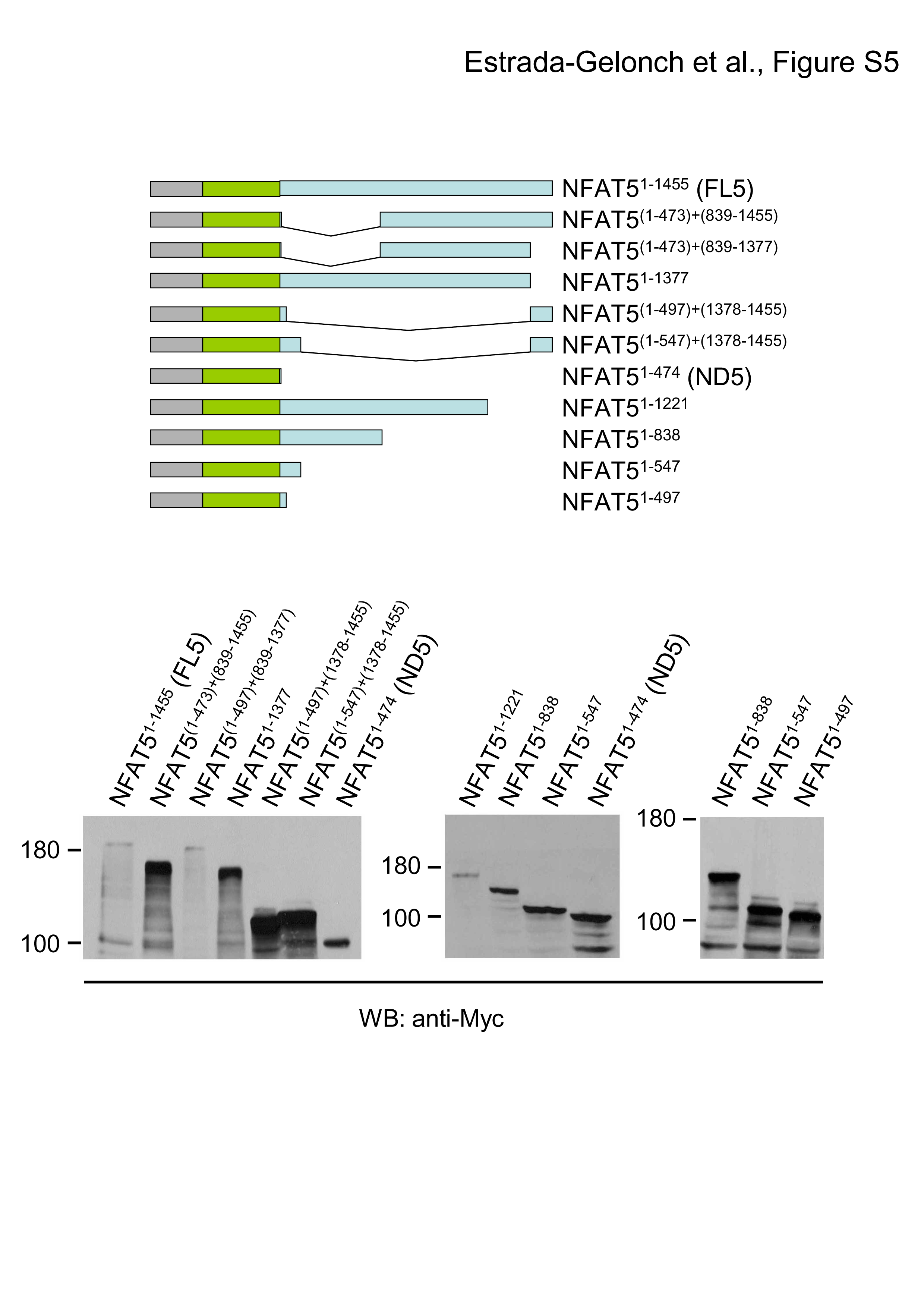

Supplement: Figure S5 — Schematic representation of NFAT5a constructs corresponding to deletions in the CTD. Constructs were tagged with 6 copies of a Myc epitope at their amino terminus and GFP at their carboxy terminus. Western blots were done with anti-Myc antibody in lysates from HEK293 cells transfected with the indicated constructs. (1.12 MB TIF) [file pone.0007036.s005.tif]
